# Assessing the inflammatory severity of the terminal ileum in Crohn disease using radiomics based on MRI

**DOI:** 10.1186/s12880-022-00844-z

**Published:** 2022-07-04

**Authors:** Honglei Ding, Jiaying Li, Kefang Jiang, Chen Gao, Liangji Lu, Huani Zhang, Haibo Chen, Xuning Gao, Kefeng Zhou, Zhichao Sun

**Affiliations:** 1 The First School of Clinical Medicine, Zhejiang Chinese Medical University, Hangzhou, People’s Republic of China; 2 Department of Radiology, The First Affiliated Hospital of Zhejiang Chinese Medical University, 54 Youdian Road, Shangcheng District, Hangzhou, 310006 People’s Republic of China; 3 Department of Gastroenterology, The First Affiliated Hospital of Zhejiang Chinese Medical University, Hangzhou, People’s Republic of China; 4 Department of Radiology, The Second Affiliated Hospital of Zhejiang University School of Medicine, Hangzhou, People’s Republic of China

**Keywords:** Crohn disease, Terminal ileum, Radiomics, Magnetic resonance imaging

## Abstract

**Background:**

Evaluating inflammatory severity using imaging is essential for Crohn’s disease, but it is limited by potential interobserver variation and subjectivity. We compared the efficiency of magnetic resonance index of activity (MaRIA) collected by radiologists and a radiomics model in assessing the inflammatory severity of terminal ileum (TI).

**Methods:**

121 patients were collected from two centers. Patients were divided into ulcerative group and mucosal remission group based on the TI Crohn’s disease Endoscopic Severity Index. The consistency of bowel wall thickness (BWT), relative contrast enhancement (RCE), edema, ulcer, MaRIA and features of the region of interest between radiologists were described by weighted Kappa test and intraclass correlation coefficient (ICC), and developed receiver operating curve of MaRIA. The radiomics model was established using reproducible features of logistic regression based on arterial staging of T1WI sequences. Delong test was used to compare radiomics with MaRIA.

**Results:**

The consistency between radiologists were moderate in BWT (ICC = 0.638), fair in edema (κ = 0.541), RCE (ICC = 0.461), MaRIA (ICC = 0.579) and poor in ulcer (κ = 0.271). Radiomics model was developed by 6 reproducible features (ICC = 0.93–0.96) and equivalent to MaRIA which evaluated by the senior radiologist (0.872 vs 0.883 in training group, 0.824 vs 0.783 in validation group, *P* = 0.847, 0.471), both of which were significantly higher than MaRIA evaluated by junior radiologist (AUC: 0.621 in training group, 0.557 in validation group, all, *P* < 0.05).

**Conclusion:**

The evaluation of inflammatory severity could be performed by radiomics objectively and reproducibly, and was comparable to MaRIA evaluated by the senior radiologist. Radiomics may be an important method to assist junior radiologists to assess the severity of inflammation objectively and accurately.

**Supplementary Information:**

The online version contains supplementary material available at 10.1186/s12880-022-00844-z.

## Introduction

Crohn’s Disease (CD) is a chronic inflammatory bowel disease with unknown etiology [1]. CD presents with periods of clinical remission and activity, persistent inflammatory activity, and is thought to trigger intestinal damage, leading to complications and affecting overall treatment [2, 3]. The terminal ileum (TI) is the most common site of CD, and previous studies have shown complications such as stenosis are common when involved [4]. Although the TI can be reached by conventional colonoscopy, complete information cannot be obtained, the use of small bowel endoscopy may compensate for this shortcoming, but complications or side effects need to be considered [5–8].

Magnetic resonance imaging (MRI) can non-invasively assess the small bowel environment and reveal the extent of extraluminal disease, therefore, it has been more frequently used than endoscopy in the clinical management of CD [9, 10]. The magnetic resonance index of activity (MaRIA), which was developed to standardize the MRI findings, has been shown to be excellent in detecting the inflammation of CD with the CDEIS as a reference standard [11, 12].

However, MaRIA results are subjective and depend on clinician experience [13]. Therefore, an accurate, reproducible and objective assessment for CD severity is of particular importance. In recent years, radiomics has been developed as a new method for reflecting the changes of diseases by mining non-visual data of images and widely validated in the field of cancer imaging [14–16]. The high-dimensional features of images may accurately and objectively reflect the manifestations of lesions. In CD, some previous studies have reported that some invisible texture features were associated with inflammatory activity of the intestinal wall or pathological markers of inflammation [17, 18]. However, its efficacy in assessing the severity of inflammation remains unclear. Therefore, this study aimed to use radiomics in detecting CD inflammatory severity and compare the MaRIA evaluated by different experienced radiologists in order to quantify the efficacy of radiomics.

## Methods and materials

### Subject selection

**Fig. 1:**
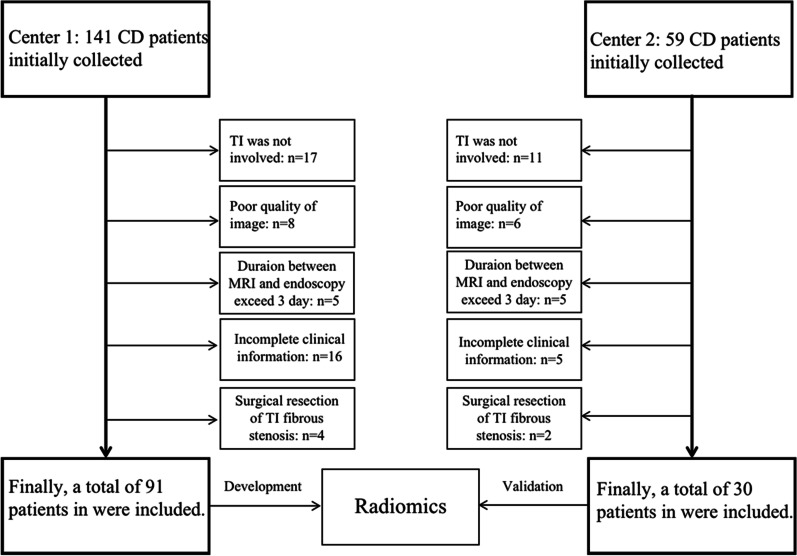
Flow diagram of the study subjects. 121 patients were included according to the included and excluded criteria. The included patients were examined by MRI and endoscopy, and had complete clinical information needed for the study

The ethics committee of our institutional approved this retrospective study (2020-KL-035-01) and the need for written informed consent was waived. Patients diagnosed with CD in two centers were retrospectively collected. All subjects were screened according to the inclusion and exclusion criteria. The inclusion criteria were as follows: (1) patients who performed ileocolonoscopy; (2) patients who underwent MRI scan. The exclusion criteria included: (1) Incomplete clinical data; (2) The interval between endoscopy and MRI was more than 3 days, in order to avoid the change of intestinal real-time state; (3) The area of interest (ROI) cannot be delineated or the TI is not clearly visible on imaging; (4) Patients whose TI was dominated by fibrous stenosis or surgically removed; (5) L2 sub-type and upper digestive tract involvement with L2 were excluded according to the Montreal Classification because of uninvolved TI, the patient excluded workflow can be seen in Fig. 1.

### MRI acquisition

All patients in two centers were imaged using a 3.0T MR (General Electric Company, USA). Patients were placed in the supine position on the examination bed. An abdominal surface coil was used to modulate the signal. Before imaging, patients were given 20 mg Raceanisodamine Hydrochloride intramuscularly to reduce bowel peristalsis and dilate the lumen fully. The scanning sequences mainly included T2-weighted fat-suppression sequences, diffusion-weighted imaging sequences (b-values included 500 and 800 mm^2^/s), and contrasted enhancement based on liver acquisition with volume acceleration (LAVA) sequence. Arterial-phase enhanced imaging based on liver acquisition with volume acceleration (LAVA) sequence was acquired 20 s after intravenous administration of 0.1 mmol/kg body weight of Gadolinium-DTPA through the cubital vein at a rate of 2.5 mL/s. The detailed parameters are listed in Table 1. Due to the application of the arterial phase in processing of radiomics, the scanning parameters of contrasted sequence in center 2 was only presented.

**Table 1:** Protocol for MR image acquisition

	Plane	TR (ms)	TE (ms)	Slice thickness (mm)	FOV	Matrix	NEX
T1WI + C (Center 1)	Axial/coronal	3.7	1.1	5	280 × 80	288 × 288	1
T1WI + C (Center 2)	Axial	3.7	1.6	5	280 × 80	–	1
T2WI-FS (Center 1)	Axial	3333.3	85.2	7	36 × 80	–	2
DWI (500/800 mm^2^/s, Center 1)	Axial	7058.8	81.8	7	36 × 80	–	4

*TR* Repetition time, *TE* Echo time, *FOV* Field of view, *NEX* Number of excitations, *T1WI* + *C* Contrasted T1-weighted image, *T2WI-FS* T2-weighted image-Fat Suppression, *DWI* Diffusion weighted imaging

### Endoscopic data

Ileocolonoscopy was used as a reference standard of inflammatory severity. All patients were given 3000–4000 mL of compound polyethylene glycol and electrolyte solution for intestinal cleansing the night before their examination, and to ensure the intestinal tract was clearly visible under endoscopy. 40 ml Simethicone Emusion was also given orally on the morning of inspection. For comparing the consistency of the MaRIA and endoscopic assessment of the ROI, the TI, which was defined as the segment of the small intestine within 10 cm of the ileocecum, was assessed by CDEIS of terminal ileum (tCDEIS) rather than the overall CDEIS score. For CDEIS calculation, the endoscopic variables were as originally defined: deep ulcers and superficial ulcers (presence or absence), ulcerated surface and affected surface (evaluated on a 10 cm linear analogue scale), ulcerated and non-ulcerated stenosis [19]. All procedures were carried out by a gastroenterologist with more than 20 years of experience using the standard equipment (CFQ240, Olympus, Japan). In CDEIS score, 3.5 and below are classified as mild inflammation with a tendency toward mucosal healing, a score of 3.5–7 is considered moderated disease and less than 7 was treated as endoscopic remission, a score more than 7 was treated as ulcerative disease [20], which indicated a poorer prognosis and moderate-high risk of clinical treatment [21]. Therefore, all patients were classified as ulcerative group (UG, tCDEIS > 7), and mucosal remission group (MG, tCDEIS ≤ 7).

### MaRIA collection

MaRIA was evaluated by MR findings including bowel thickness, relative contrast enhancement (RCE = ((WSI postgadolinium − WSI pregadolinium)/(WSI pregadolinium)) × 100 × (s.d. noise pregadolinium/s.d. noise postgadolinium))), edema (hyperintensity on T2-weighted sequence relative to the signal of the psoas muscle) and ulcers (defined as deep depressions in the mucosal surface) [10] to reflect the effectiveness of the radiologist's assessment. Two radiologists, one with 15 years of experience in abdominal imaging (ER1) and another with 5 years of experience in abdominal imaging (ER2) were asked to calculate the score in the thickest region of the bowel wall according to these following formulas: MaRIA = 1.5 × bowel thickness + 0.02 × RCE + 5 × edema + 10 × ulcer. The two radiologists were blind with each other and the endoscopic results.

MaRIA less than 7 indicates normal mucosa, a score of 7–11 is considered mild disease, a score greater than 11 indicates ulcerated lesions [11], and MaRIA less than 11 was treated as ulcer healing. To match the classification of tCDEIS, the cut-off value of MaRIA was set to 11 (less than 11 was classified as MG and greater than 11 was classified as UG).

### Radiomics processing

**Fig. 2:**
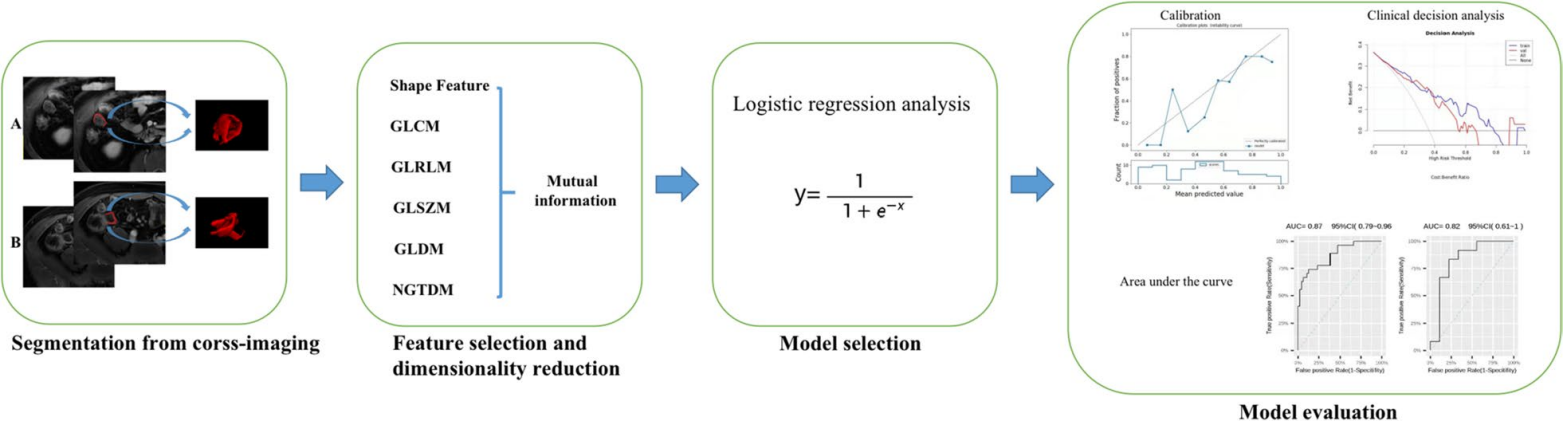
The workflow of Radiomics analysis procedures. Step 1 Segmentation from cross-image: **A** represent the outline of MG, a 34-year-old male diagnosed A2B1L1 according to Montreal classification (tCDEIS = 4.5); **B** presents the outline of UG, a 65-year-old woman diagnosed A3B2L3 according to Montreal classification (tCDEIS = 7.5). Step 2 Feature selection and screening: Feature types include shape features, GLCM, GLDM, GLRLM, GLSZM, NGTDM, and mutual information was selected for dimensionality reduction. Step 3 The modeling approach: Logistic analysis was selected to model the retained features. Step 4 Model assessment: ROC curve, calibration curve and clinical decision curve were selected for assessing the efficiency of the radiomics model. (*Note*: GLCM = Gray Level Co-occurrence Matrix, GLRLM = Gray Level Run Length Matrix, GLSZM = Gray Level SizeZone Matrix, GLDM = Gray Level Dependence Matrix, NGTDM = Neighborhood Gray-Tone Difference Matrix)

The arterial stage of T1 weighted enhanced imaging of all patients was imported into ITK-SNAP (Version 3.8.0, www.itksnap.org) software [22] in DICOM format. Two radiologists who evaluated MaRIA performed outlining along the TI wall on the axial image slice by slice manually. The lumen of intestinal was excluded and the intestinal wall was not clearly displayed or the visual overlap of adjacent intestinal walls were not included in the ROI. Part of the outlining results were shown in Fig. 2A, B.

All the outlining images and the corresponding primary images were imported into Dr. Wise Multimodel Research Platform (Version1.6.3.6 Deepwise&League of PHD Technology Co., Ltd, Beijing, China), which has been reported by previous study [23, 24] for one-to-one matching, labeling and ROI features extraction. 1648 features were selected, including shape feature, Gray Level Co-occurrence Matrix (GLCM), Gray Level Run Length Matrix (GLRLM), Gray Level SizeZone Matrix (GLSZM), Gray Level Dependence Matrix (GLDM) and Neighborhood Gray-Tone Difference matrix (NGTDM), all these features were transformed according to LoG, Square, Square Root, Logarithm, Gradient, Wavelet and local binary pattern (LBP). Detailed mathematical definitions of features were provided in the Additional file1. All patients in center 1 were randomly divided into the training group and the validation group in a ratio of 7:3 and underwent 5 cycles. Before feature dimension reduction, the features with a miss rate greater than 10% was selected to be cleared, and a feature that contributed less to the label classification will be removed when correlation coefficient greater than 0.9 between features to avoid redundancy. Mutual information (MI) method was selected to dimensionality reduction of features and logistic model was selected for developed radiomics model. The workflow in summary can be seen in Fig. 1. The patients in center 2 was used to validate the model as an external cohort.

### Calibration and clinical utility of radiomics model

Calibration curve was used to measure how well a probabilistic prediction of an event matches the true underlying probability of the event. Decision curve analysis was used to measure the clinical efficacy of Radiomics model; A decision analysis measure, called the net benefit of the model, was calculated for the possible threshold probabilities. The benefits (proportion of true positives) and disadvantages (proportion of false positives) are added, and the diagnosis is weighted by the relative harm of false positives and false negative results. The net benefit values of the diagnostic model were standardized for prevalence.

### Statistical analysis

The statistical analyses about clinical characteristics and assessment by radiologists based on MRI were performed using SPSS software (version 22.0) and MedCalc software (version 15.2). The consistency of image sketching, dimension reduction of features and machine learning model construction were carried out on Dr. Wise Multimodel Research Platform mentioned above. Descriptive statistics were performed for part of the patient clinical data. The data conforming to normal distribution were represented by Mean ± standard deviation (SD), and the difference was calculated by independent sample t-test. Chi-square test or Fisher's exact test were used for counting data. Median (interquartile range) was performed to describe the distribution of data, such as tCDEIS, BWT, RCE, and MaRIA. The intraclass correlation coefficient (ICC) test and weighted Kappa coefficient were provided to test the consistency in measurements and features of ROI between radiologists. Area under the receiver operating curve (AUC), sensitivity and specificity were reported for presenting effectiveness of MaRIA evluated by radiologists, Delong test used to measure statistical differences between MaRIA and radiomics. A p-value less than 0.05 was considered statistically significant. Reliability was constitute “poor,” “fair,” “moderate,” and “good” reliability, with a corresponding cutoff value of ICCs or κ value of < 0.4, 0.41–0.6, 0.61–0.8, and > 0.8, and higher than 0.9 was considered the good agreement in ROI.

## Results

### Patient characteristics

A total of 200 patients (141 between January 1, 2016 and April 30, 2020 from Center 1, 59 between May 1, 2019 and July 1, 2021 from Center 2) were studied and 121 patients (91 in Center 1, 30 in Center 2). 68 patients (52 in Center 1, 16 in Center 2) were classified as the UG and tCDEIS in Center 1 was 10,00 (IQR 2.50), Center 2 was 9.00 (IQR 2.00). 53 patients (39 in Center 1, 14 in Center 2) were grouped in MG, the tCDEIS in in Center 1 was 3.50 (IQR 1.50) and 3.25 (IQR 2.00) in Center 2. CRP and ESR were elevated relative to reference values (normal ≤ 8 mg/l, ≤ 7.2 mm/L) in 35 and 31 patients, respectively. There was significant difference in gender and CRP, but no significant difference in average age, surgery, the history of perianal involvement, ESR and treatment measures in two groups (Table 2).

**Table 2:** Clinical and biological characteristics

	UG	MG	*P*
Number	68	53	
Average age/years, (Mean ± SD)	40.2 ± 16.2	34.1 ± 13.6	0.07
Female, n (%)	13 (19.1%)	26 (49.1%)	< 0.01
History of Perianal involvement, n (%)	15 (22.1%)	15 (28.3%)	0.43
History of surgery, n (%)	20 (33.3%)	15 (32.4%)	0.89
Inflammatory biomarkers, n (%)			
ESR (> 7.2 mm/L)	13 (19.1%)	18 (34.0%)	0.09
CRP (> 8 mg/L)	14 (20.6%)	21 (39.6%)	0.02
Treatment measures, n (%)			0.22
Anti-TNF antibodies	32 (47.1%)	17 (32.1%)	
Immunosuppressant	18 (26.5%)	14 (26.4%)	
Hormone steroid	1 (1.5%)	3 (5.7%)	
Combination of two or more drugs	17 (25.0%)	19 (35.8%)	
tCDEIS(IQR, Center1)	10,00 (2.50)	3.50 (1.50)	–
tCDEIS(IQR, Center2)	9.50 (2.00)	3.25 (2.00)	–

*UG* Ulcerative group, *MG* Mucosal remission group, *ESR* Erythrocyte sedimentation rate, *CRP* C-reactive protein, *tCDEIS* Crohn’s Disease Endoscopic Index of Severity of terminal ileum

### MaRIA and relative parameters measurement

Since the non-contrast sequence was not performed in Center 2, the MaRIA and related parameters of Center 2 were not calculated. The median value of radiologists in detecting BWT was 4.35 mm (IQR 1.52), 3.52 mm (IQR 1.39), respectively. The measurements of RCE between observers were 128.00 (IQR 75.38) and 70.00 (IQR 52.75), respectively. A moderate consistency of BWT between radiologists were detected (ICC = 0.638 Table 3), fair consistency was detected in parameters of RCE, edema reading (ICC = 0.461, κ = 0.541, respectively, Table 3) and poor in ulcer (κ = 0.271) between radiologists.

**Table 3:** Measurements between radiologists

	ER1	ER2	ICC/κ
BWT (Median, IQR)	4.35 (1.52)	3.52 (1.39)	0.638
RCE (Median, IQR)	128.00 (75.38)	70.00 (52.75)	0.461
MaRIA (Median, IQR)	9.50 (4.61)	7.12 (3.59)	0.579
Edema, n (%)	11 (12.1)	4 (4.4)	0.541*
Ulcer, n (%)	6 (6.6)	1 (1.1)	0.271*

*ER1* Experienced radiologist 1, *ER2* Experienced radiologist 2, *RCE* Relative contrast enhancement, *MaRIA* magnetic resonance index of activity, *IQR* Interquartile range, *ICC* Intraclass correlation coefficient

*Weighted Kappa coefficient

**Fig. 3:**
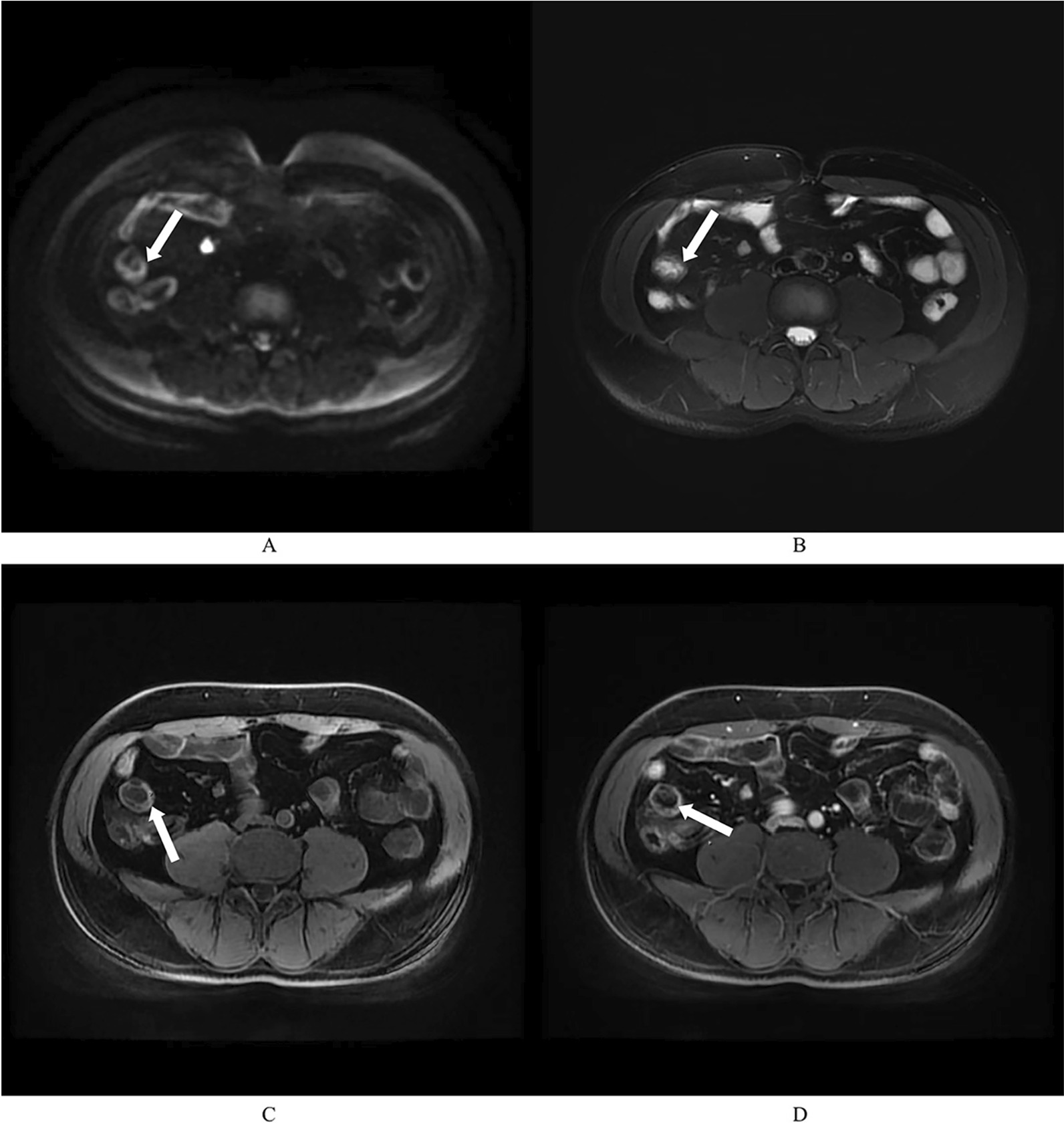
The presentation of MaRIA assessment by two radiologists. A 49-year-old male patient with Montreal classification of A3B1L1 was classified UG (tCDEIS = 8). **A** and **B**, axial DWI sequence (**A**) and axial T2-weighted single-shot fast spin-echo image with fat saturation (**B**) presented the abnormality of terminal ileum (arrow). **C** and **D** represent pre- and post-enhancement sequences, and the enhancement could be detected of the intestinal wall (arrow). The BWT measured by R1 (the senior) and R2 (the junior) was 4.75 mm, 4.00 mm, respectively, and the RCE assessed by R1 and R2 was 119.7, 93.6 separately. R1 considered that the edema could be detected, while R2 did not. No sign of ulcer was reported in both radiologists. MaRIA evaluated by R1 was 14.52, and 7.87 by R2. Obviously, R1 made an accurate assessment and R2 made an inappropriate diagnosis

MaRIA score measurement between investigators was 9.50 (IQR 4.61) and 7.12 (IQR 3.59). Obviously, fair agreement was detected between two radiologists (ICC = 0.579, Table 3). Part of the evaluation process and results can be obtained in Fig. 3.

### Features retained and model constructed

An ICC higher than 0.9 was considered to be retained for dimensionality reduction. After screening, a total of 1229 features were considered to have high consistency. 5 basic clinical information features were removed and 1224 were retained for reduction. 6 highly consistent parameters (ICC 0.93–0.96) were obtained after dimensionality reduction by MI method eventually.

Logistic regression is used to constructed machine learning models based on retained features with the forward method, and the results can be obtained in Table 4. The Radscore was calculated by the following formula:

$$\begin{aligned} & - 0.{6136 } \times {\text{ Wavelet}} - {\text{LHL}}\_{\text{firstoeder}}\_{\text{Maximum}} \\ & + \, 0.{5292 } \times {\text{ Wavelet}} - {\text{LLL}}\_{\text{firstorder}}\_{\text{Minimum}} \\ & + \, 0.0{128 } \times {\text{ Wavelet}} - {\text{HHL}}\_{\text{firstorder}}\_{\text{Skewness}} \\ & - \, 0.0{623 } \times {\text{ Wavelet}} - {\text{HHH}}\_{\text{gldm}}\_{\text{DependenceVariance}} \\ & + \, 0.{3}0{3}0 \, \times {\text{ Wavelet}} - {\text{LLL}}\_{\text{glrlm}}\_{\text{RunVariance}} \\ & - \, 0.{3754 } \times {\text{ Log}} - {\text{sigma}} - {2} - 0 - {\text{mm}} - {\text{3D}}\_{\text{SmallAreaEmphasis}} \\ & + \, 0.{1135} \\ \end{aligned}$$

**Table 4:** The results of multivariate logistic regression for the retained features

Retained features	Coefficient	OR	*P*
Wavelet-LHL_firstoeder_Maximum	− 0.6136	0.541	0.007
Wavelet-LLL_firstorder_Minimum	0.5292	1.698	0.004
Wavelet-HHL_firstorder_Skewness	0.0128	1.013	0.034
Wavelet-HHH_gldm_DependenceVariance	− 0.0623	0.940	0.014
Wavelet-LLL_glrlm_RunVariance	0.3030	1.354	0.009
Log-sigma-2-0-mm-3D_SmallAreaEmphasis	− 0.3754	0.687	0.009

*OR* Odds ratio, *GLDM* Gray level dependence matrix, *GLRLM* Gray level run length matrix

### Models assessment for identifying inflammatory severity

The MaRIA was modeled to represent the radiologists’ assessment of inflammatory severity based on MR findings. When evaluating the MaRIA acquired by ER1, the AUC was 0.883, with a sensitivity and specificity of 0.857 and 0.909 in the training group, respectively. The AUC, sensitivity and specificity of the testing group were 0.783, 0.700, and 0.867, separately. The model performance of ER2 had an AUC of 0.621, with a sensitivity and specificity of 0.685 and 0.577 in the training group, respectively. The AUC in the testing group was 0.557, the sensitivity and specificity was 0.548 and 0.603, separately.

**Fig. 4:**
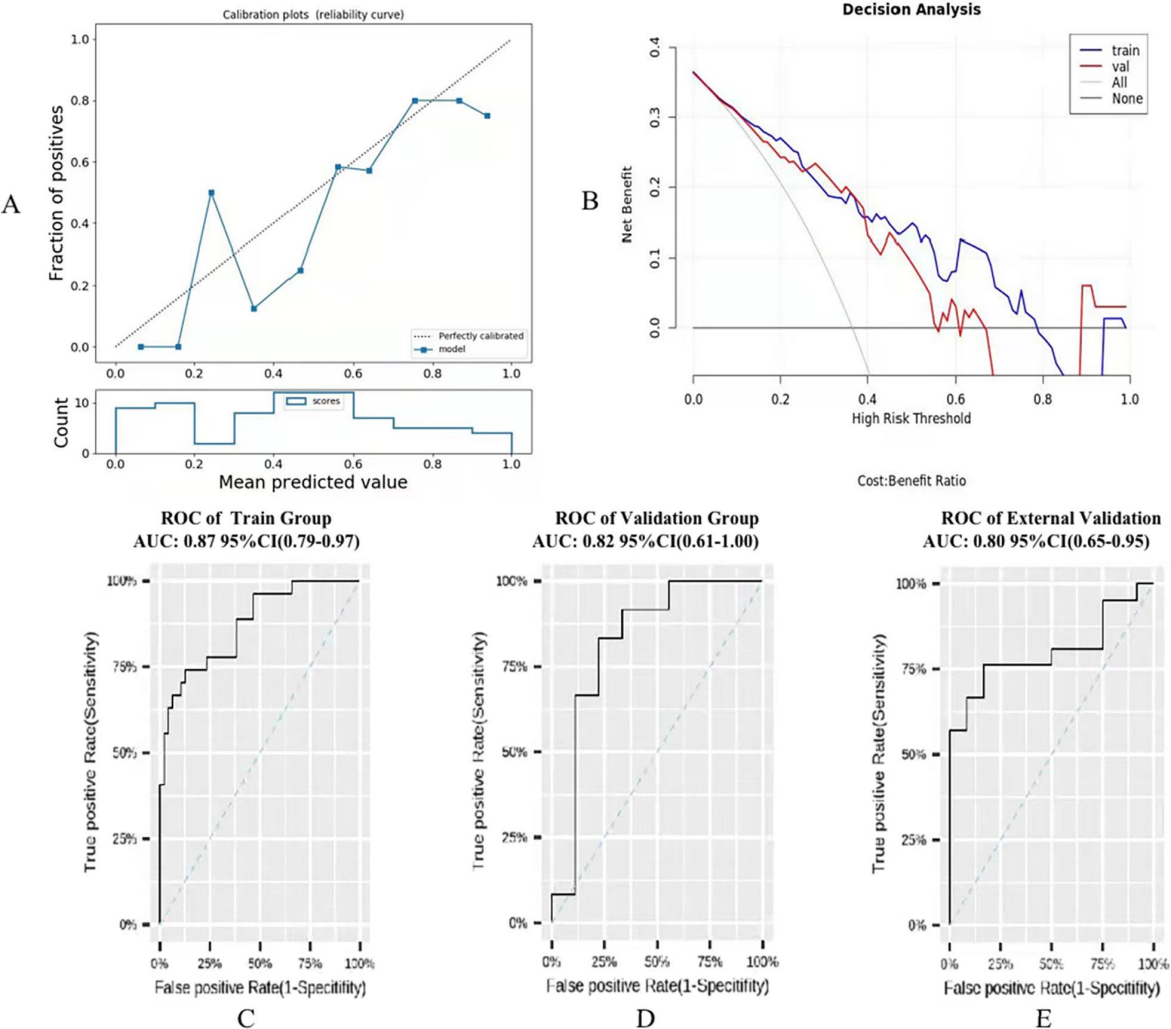
The output of Radiomics model in assessing the inflammatory severity of TI (**A**–**E**). **A** presents the reliability curve; **B** presents the clinical decision curve, when the risk threshold range is 0.1–0.65, the model presents the benefit; **C** represents the result of training group. **D** displays the result of validation group, and **E** shows the results of the external validation group

The radiomics model has been established to distinguish the state of ROI depending on the reserved features by logistic regression analysis. In training group, the AUC, sensitivity and specificity were 0.872, 0.741 and 0.809, respectively (Fig. 4C). The AUC of the validation group was 0.824, sensitivity and specificity were 0.833 and 0.619, respectively (Fig. 4D). The extenal validation group of center 2 proved the stability of the model with an AUC, sensitivity and specificity of 0.800, 0.729 and 0.731, separately (Fig. 4E). The calibration curve showed a good match between the prediction of image model and the real situation (Fig. 4A), and the clinical decision curve presented that the model shows benefits within a certain risk range (Fig. 4B). All specific parameters about the radiomics model can be found in Table 5. Delong test was found that the classifying efficiency of radiomics was equivalent to that of the senior radiologist (*P* = 0.847, 0.471, respectively, Table 6), both of which were higher than that of junior radiologist, and the difference was statistically significant (both, *P* < 0.05, Table 6).

**Table 5:** The specific parameters of radiomics model

	Precision	AUC (95%CI)	Sensitivity	Specificity	F1-score	Recall	PPV	NPV
Training	0.784	0.872 (0.789–0.965)	0.741	0.809	0.714	0.741	0.690	0.844
Validation	0.762	0.824 (0.612–1.000)	0.667	0.889	0.762	0.667	0.889	0.667
Ex-val	0.727	0.800 (0.649–0.954)	0.729	0.731	0.744	0.762	0.727	0.546

*EX-val* External validation**,** *AUC* Area under the ROC curve, *PPV* Positive predictive value, *NPV* negative predictive value

**Table 6:** The results of Delong test among different models

	Training group	Validation group
ER1 versus Radiomics	0.847	0.471
ER2 versus Radiomics	0.015*	0.002*
ER1 versus ER2	0.008*	0.042*

*Presents the statistically significant of results

## Discussion

In this present study, we found that inflammatory severity in CD can be resolved from MR studies by the radiomics objectively and repeatedly. Moreover, the method has a comparable ability with MaRIA as evaluated by our senior radiologist.

MRI, as a non-invasive method with good soft tissue resolution and no radiation exposure, plays an important role in the management of CD. In this technique, quantitative indicators of inflammation, such as MaRIA was established based on morphological and imaging parameters. However, due to the complexity of intestinal anatomy, high consistency of these features were difficult to guarantee among radiologists. Some studies have reported highly variation in assessing MRI features among radiologists [13, 25, 26]. In the reading of RCE, agreement was only moderate between senior radiologists (ICC = 0.55), and even lower when combined with junior radiologists (ICC = 0.42) according to Tielbeek et al. [13], which was similar to the results of a recent central reading (the ICC of RCE was 0.59) [26]. Interestingly, the consistency between the two senior radiologists was lower than the four radiologists (including two junior radiologists) as a whole (κ value 0.57 vs 0.66) in the evaluation of edema parameters, suggesting that subjectivity may have played a role in this evaluation. In terms of ulcer reading, the consistency reported by current researches are pessimistic, no consistency was detected by Tsai et al. [27] and only poor consistency was reported by Rees et al. [28]. The results of this study were similar to those previous studies, and no good agreement was obtained in the reading of MRI features between radiologists. This may limit the flexible application of MaRIA among radiologists, previous studies have reported the effectiveness of MaRIA in assessing the severity of terminal ileum/small intestinal inflammation by different radiologists at different centers, with variable AUC range of 0.741–0.92 [29–32]. Although these MRI imaging features have been standardized to ensure reading consistency among radiologists in recent years, the issue still exist and has been reported in recent study [33], especially among junior radiologists.

Computer-assisted image analysis overcame the subjectivity and improved the reproducibility of findings [34–36]. Radiomic technology allows for a high level of diagnostic accuracy by detecting features smaller than the human eye can discern on MRI. With the addition of machine learning, radiomics technologies have recently garnered strong attention from the scientific community [37–40]. In terms of CD, part of researches [34, 41, 42] confirmed that it can improve the repeatability and agreement of measurements. Moreover, some previous studies have found correlations between some texture features and the pathological mechanisms of inflammation [17, 18]. Therefore, image analysis can be an effective method for inexperienced radiologists to improve the ability to assess CD.

In this study, features with good reproducibility were retained based on MRI, most of which were Wavelet-transformed features, prior studies have suggested that it may reveal heterogeneity in the ROI and suggested a poorer prognosis [43], therefore, it may be an image-based indicator of more severe inflammation or poorer clinical outcome in patients with CD [21]. In addition, "skewness" features based on wavelet transform was retained, a texture analysis study found that skewness is related to angiogenesis [18], a confirmed and important pathological marker of inflammation in CD [44], and Makanyanga et al. [17] further confirmed that that the weight of "skewness" feature was related to the enhancement degree of the ROI on MR images, therefore, this type of feature may be an alternation of RCE measured manually by radiologists, which presented only fair consistency in this study between radiologists. However, the specific relationship between retained features and the pathological mechanism of inflammation has not been disclosed in this study and further confirmation is needed.

Finally, we modeled the radiomics by retained features and compared the efficiency of MaRIA**.** The diagnostic efficacy of the radiomics study were in parallel with senior radiologists who were limited supply, and better than that of junior radiologists. Although subjectivity is also unavoidable when sketching ROI, retained characteristics are highly consistent between radiologists, further preventing the effect of subjectivity. The results of this study suggest that Radiomics could assess the severity of inflammatory steadily and accurately, and has applications in the clinical realm.

There are several limitations of this study. Firstly, our sample size is relatively small in our research center, most subjects preferred clinical follow-up rather than hospitalization, therefore, MRI or endoscopic data may be absent or incomplete. More samples and multicenter study need to be performed. Secondly, we choose the terminal ileum as the ROI, the initial and most involved site of CD, not global involvement of the intestinal tract. Thirdly, since terminal ileum lesions may be discontinuous or asymmetric, accurate point-to-point endoscopic matching is required in the future. Fourthly, tCDEIS and CDEIS are slightly different. tCDEIS is the CDEIS score that only includes the terminal ileum, while CDEIS is to calculate the total score of affected segments and divide by the number of affected segments to obtain an average value. CDEIS may be slightly higher or lower than tCDEIS, depending on the severity of the colonic lesions.

## Conclusions

In summary, while MaRIA is a good criterion for grading CD inflammation, it is subjective and depends heavily on the experience of the radiologist. The radiomics based on MRI imaging features was objective and reproducible. Our study along this direction has revealed that the radiomics model performs similarly to senior radiologists and may be an important method to assist junior radiologists in assessing severity of inflammation in the terminal ileum.

## Supplementary Information

**Additional file 1.** Mathematical definition of radiomic feature extraction and Mutual information (MI).

## Data Availability

The data generated and analyzed during the current study are not publicly available but are available from the corresponding author on reasonable request.
