# Supplementary material for: Assessing the inflammatory severity of the terminal ileum in Crohn disease using radiomics based on MRI
Source: BMC Med Imaging. 2022 Jul 4;22:118. doi: 10.1186/s12880-022-00844-z (PMC9254684; doi:10.1186/s12880-022-00844-z)
Supplement: Supplementary file 1 — Additional file 1. Mathematical definition of radiomic feature extraction and Mutual information (MI). [file 12880_2022_844_MOESM1_ESM.docx]

**Supplementary material**

**Mathematical definition of radiomic feature extraction:**

Briefly speaking, the extracted radiomics features included 26 shape feature, describing the two-dimensional size, three-dimensional size, and shape of ROI (eg, Minor Axis Length or Sphericity), these features are independent of the gray-scale intensity distribution of ROI and are therefore only computed on non-derived images and masks [1]; 24 gray-level co-occurrence matrix (GLCM) features [1], describing how often a voxel occurs horizontally, vertically, or diagonally to adjacent with similar gray-level value (eg, Cluster prominence or Cluster tendency); 14 gray-level dependence matrix(GLDM) [2], describing number of times a voxel with gray level with dependent voxels in its neighbors appears in image (eg, Small dependence emphasis or Gray level non-uniformity); 16 gray-level run length matrix (GLRLM) features [3], describing how many voxels with a given gray-level value occur in a sequence in the given direction (eg, short run emphasis or long run emphasis); 16 gray-level size zone matrix (GLSZM) features [4], describing how many voxels with a given graylevel value are connected (eg, small area emphasis or large area emphasis); 5 neighborhood gray-tone difference matrix (NGTDM) features，describing the difference between a voxel and the average gray-level value of its neighbors (eg, coarseness or busyness) .

**Mutual information (MI) in mathematic definition:**

We used mutual information(MI) to determine the best subset of radiomic features in identifying the severity of terminal ileum inflammation. MI is an algorithm that measures the degree of similarity between two different labels of the same data, regardless of the absolute value of the label: the arrangement of class or cluster label values does not change the score value in any way. When extracting features from classification problems, mutual information can be used to measure the correlation between a feature and a specific category, which is different from the correlation coefficient. If the amount of information is larger, the correlation between feature and this category will be larger. The reverse is also true[6-7].

Mathematically, the MI is expressed as:

$MI\left( U, V \right)=\sum_{i=0}^{\left| U \right|} \sum_{j=1}^{\left| V \right|} \frac{\left| U_{i} \right|\cup\left| V_{j} \right|}{N}\log\frac{N\left| U_{i} \right|\cup V_{j}}{\left| U_{i}V_{j} \right|}$

U_i_ is the number of samples in the U cluster, V_j_ is the number of samples in the V cluster.

**Supplementary reference**

[1] Zwanenburg A, Leger S, M Vallières, et al. Image biomarker standardisation initiative[J]. Radiotherapy & Oncology, 2016. DOI: 10.1016/S0167-8140(18)31291-X

[2] Sun C, Wee W G. Neighboring gray level dependence matrix for texture classification[J]. 1983, 23(3):341-352. DOI: 10.1016/0734-189x(83)90032-4

[3] Galloway M. Texture analysis using gray level run lengths[J]. Computer Graphics & Image Processing, 1975, 4( 2):172-179. DOI: 10.1016/S0146-664X(75)80008-6

[4] Thibault G, Fertil B, Navarro C, et al. Texture Indexes and Gray Level Size Zone Matrix Application to Cell Nuclei Classification[C]// 10th International Conference on Pattern Recognition and Information Processing. 2009.

[5] Amadasun M, King R. Textural features corresponding to textural properties[J]. IEEE Transactions Systems Man & Cybernetics, 1989, 19(5):1264-1274. DOI: 10.1109/21.44046

[6] Guo B, Nixon M S. Gait Feature Subset Selection by Mutual Information[J]. IEEE Transactions on Systems Man & Cybernetics Part A Systems & Humans, 2008. DOI: 10.1109/TSMCA.2008.2007977

[7] Zaffalon M, Hutter M. Robust Feature Selection by Mutual Information Distributions[J]. 2014.
